# Apathy in Frontotemporal Degeneration: Neuroanatomical Evidence of Impaired Goal-directed Behavior

**DOI:** 10.3389/fnhum.2015.00611

**Published:** 2015-11-10

**Authors:** Lauren Massimo, John P. Powers, Lois K. Evans, Corey T. McMillan, Katya Rascovsky, Paul Eslinger, Mary Ersek, David J. Irwin, Murray Grossman

**Affiliations:** ^1^Department of Neurology, Frontotemporal Degeneration Center, Perelman School of Medicine, University of PennsylvaniaPhiladelphia, PA, USA; ^2^School of Nursing, University of PennsylvaniaPhiladelphia, PA, USA; ^3^Department of Neurology, Penn State Hershey Milton S. Hershey Medical CenterHershey, PA, USA

**Keywords:** frontotemporal degeneration, MRI, DWI, executive function, motivation, apathy

## Abstract

**Background:** Apathy, the major manifestation of impaired goal-directed behavior (GDB), is the most common neuropsychiatric syndrome associated with behavioral variant frontotemporal degeneration (bvFTD). The behavioral and biological mechanisms of apathy, however, are not well understood. We hypothesized that GDB has multiple components—including at least initiation, planning and motivation—and that GDB is supported by a network of multiple frontal brain regions. In this study, we examined this hypothesis by evaluating the selective breakdown of GDB in bvFTD, and relating these deficits to gray matter (GM) atrophy and white matter (WM) integrity.

**Methods:** Eighteen apathetic bvFTD participants and 17 healthy controls completed the Philadelphia Apathy Computerized Test (PACT). This test quantifies each of three components of GDB hypothesized to contribute to apathy. We then used regression analyses to relate PACT scores to GM atrophy and reduced white matter (WM) fractional anisotropy (FA) in bvFTD.

**Results:** Compared to controls, bvFTD participants demonstrated significant impairments in each of the three hypothesized components of GDB that contribute to apathy. Regression analyses related each component to disease in specific GM structures and associated WM tracts. Poor initiation thus was related to GM atrophy in anterior cingulate and reduced FA in the cingulum. Planning impairment was related to GM atrophy in dorsolateral prefrontal cortex and reduced FA in superior longitudinal fasciculus. Poor motivation was related to GM atrophy in orbitofrontal cortex (OFC) and reduced FA in uncinate fasciculus (UNC).

**Conclusions:** bvFTD patients have difficulty with initiation, planning and motivation components of GDB. These findings are consistent with the hypotheses that GDB encompasses at least three processes, that these are supported by a large-scale neural network within specific portions of the frontal lobe, and that degradation of any one of these prefrontal regions in bvFTD may contribute to apathy.

## Introduction

The word *apathy* derives from the Greek word *pathos* or passion. It describes a state of indifference or inertia (Robert et al., 2009). Over time the concept of apathy has undergone changes in meaning, and remains vaguely defined and broadly applied (Chase, 2011). Sometimes described as a symptom of other disorders such as depression, Marin clarified the concept of apathy for medical purposes by proposing to define apathy as a lack of motivation (Marin, 1990). One caution with Marin's definition is that “lack of motivation” may not be the only mechanism that contributes to apathetic behavior. For example, others have noted that apathy is synonymous with poor initiation (Dujardin et al., 2007). Here we adopt the definition proposed by Levy and DuBois who define apathy as the quantitative reduction of self-generated voluntary and purposeful goal-directed behavior (GDB) (Levy and Dubois, 2006). GDB is an essential facet of day-to-day human functioning. GDB allows a person to direct purposeful behavior toward a desirable goal or away from an undesirable outcome (Geurts and de Wit, 2013). While approaches emphasize a single, critical characteristic of apathy such as “lack of motivation” (Marin, 1996; Marin and Wilkosz, 2005), we hypothesize that GDB is a multi-component process that includes at least initiation, planning, and motivation. In this study, we examine deficits in these components of GDB in patients with the behavioral variant of frontotemporal degeneration (bvFTD).

Central to the concept of GDB is the integration of multiple processes that influence a person to act. We hypothesize that at least three components may contribute to GDB. *Initiation* refers to one's ability to self-generate or activate actions. The failure to execute behavior leads to apathy when processing is unable to generate a signal significant enough to initiate a response. *Planning* is the ability to elaborate plans of action. This describes high-dimensional cognitive processes in executive function that are needed to formulate and carry out complex and multi-step goals. Finally, rewards and avoidance of negative consequences or “punishment” constitute fundamental motivational functions that are based in part on the processing of rewarding and punishing information (Schultz et al., 2000). *Motivation* thus refers to the ability to associate affective (positive or negative) signals with value in performing actions.

From the perspective of our hypothesized model of GDB, apathy arises when any one of these three processes is impaired. Although each step may be necessary to achieve GDB, clinical observations of patients with neurodegenerative diseases (ND) suggest that these processes may be somewhat dissociable. For example, patients who have impairments in executive abilities needed to carry out plans of action may not find it difficult to initiate a single, simple action. Other patients may not be motivated to perform an action even though an action can be initiated. Finally, some patients may be relatively incapable of initiating an action. Therefore, we hypothesize that each of these GDB processes may be partially independent and, when any one of these is compromised, apathy may become evident.

A large-scale neural network is thought to support GDB mechanisms such as these that contribute to apathy by involving brain regions that are implicated in each of these processes (Levy and Dubois, 2006). We focus on three functional neuroanatomic regions in the frontal lobe that together appear to capture the information from internal and external environments that are likely important to GDB. This includes anterior cingulate, dorsolateral prefrontal cortex, and orbitofrontal cortex. Anterior cingulate disease thus has been associated with difficulty initiating activities (Kotchoubey et al., 2003), dorsolateral prefrontal cortex appears to contribute to the generation of higher-level planning and organization (Kaller et al., 2011), and orbital frontal cortex has been implicated in motivation (Hare et al., 2010).

Apathy is a prevalent behavioral symptom in neurodegenerative disorders (Mega et al., 1996; Clarke et al., 2008). In this study, we examined dissociable behavioral and neuroanatomic components encompassed by a multi-component model of GDB by examining apathy in patients with bvFTD (Diehl-Schmid et al., 2006). bvFTD is an ND that mainly affects the frontal and temporal lobes of the brain. This condition affects individuals at a young age, typically presenting in the fifth or sixth decade of life (Rosso et al., 2003; Massimo and Grossman, 2008). Clinically, bvFTD presents with difficulty regulating social behaviors and a profound loss of insight (Rascovsky et al., 2011). One large autopsy-confirmed study examined the frequency of behavioral symptoms in bvFTD, and found apathy was the most frequent, occurring in 84% of patients (Rascovsky et al., 2011).

Most studies of bvFTD have assumed that apathy is a single, undifferentiated behavioral phenomenon. Using such a unitary model, apathy in bvFTD has been linked to a single frontal area in prior work, including dorsolateral, anterior cingulate, or orbital frontal regions (Rosen et al., 2005; Zamboni et al., 2008; Massimo et al., 2009). Heterogeneous findings such as these may instead reflect that apathy is multi-factorial, consistent with the GDB model, and that disease in any one of these anatomic regions may compromise GDB in bvFTD and result in apathy. The Philadelphia Apathy Computerized Test (PACT) is a novel reaction time test designed to objectively measure three components of GDB that are hypothesized to contribute to apathy, including initiation, planning, and motivation. We further hypothesize that disease in specific frontal regions may compromise GDB and lead to apathy. In the present study, we relate patterns of behavioral impairment on each component of our novel measure to MRI regions of gray matter (GM) atrophy and white matter (WM) integrity in bvFTD.

## Materials and methods

### Participants

Eighteen patients (5 females) were recruited from the outpatient clinic of the Department of Neurology, University of Pennsylvania and evaluated by experienced cognitive neurologists (DJI, MG) using published consensus criteria for the diagnosis of probable bvFTD (Rascovsky et al., 2011). We focused particularly on bvFTD because apathy is very common in this condition, these patients do not have physical limitations that can confound the quantitative assessment of reduced GDB, and there are no language or visuospatial deficits that can potentially limit the interpretation of bvFTD patient performance. All patients had mild disease (MMSE ≥ 20) to minimize potential confounding factors related to severe cognitive impairment. Medical and psychiatric causes of dementia were excluded by clinical exam and blood and brain imaging tests. We also excluded individuals with depression using the Geriatric Depression Scale-Short Form (Sheikh and Yesavage, 1980) scores > 5, as depression can be confused with apathy, and we excluded participants taking benzodiazepines and other soporific medications because of their potentially sedating side effects. All participants had apathy as determined by a Neuropsychiatric Inventory (NPI) (Cummings et al., 1994) frequency by severity (FxS) score ≥ 1. The FxS score is rated on the basis of scripted questions administered to the patient's caregiver, yielding a maximum score of 12. The caregiver also rates his/her own levels of distress for each domain. Seventeen healthy, seniors served as a control group for the behavioral measure. Control participants were demographically comparable to bvFTD participants for age and education, and they self-reported a negative neurological and psychiatric history. Table 1 summarizes demographic characteristics. All participants and responsible caregivers participated in an informed consent procedure approved by the University of Pennsylvania Institutional Review Board.

**Table 1:** **Mean (±S.D.) Demographic and clinical features of patients with behavioral variant frontotemporal degeneration and healthy controls**.

	**Behavioral controls (*n* = 17)**	**Imaging controls (*n* = 24)**	**bvFTD (*n* = 18)**
Age (Years)	67.12 ± 10.82	60.71 ± 6.9	61.00 ± 5.2
Education (Years)	15.35 ± 2.91	15.79 ± 1.9	17.00 ± 3.1
Disease duration (Years)	na	na	3.70 ± 1.63
Gender (M/F)	10/7	16/8	12/6
MMSE (max score = 30)	29.47 ± 0.87	29.10 ± 1.0	27.33 ± 2.2

### Behavioral measures

#### The philadelphia apathy computerized test (PACT)

The PACT was developed to quantify components of GDB. It was developed based on a review of experimental paradigms in the literature and clinical observations of apathy (Jenkins et al., 2000; Ruh et al., 2010). Briefly, a computerized reaction time was obtained to assess initiation, planning and motivation components of GDB. There was a brief practice period of several trials for each of the measures described below, and all participants appeared to understand the tasks.

To assess the *initiation* component, the participant begins a trial by depressing and holding a designated “start” key on a computer keyboard, then a central visual stimulus (triangle) appears on the computer screen (latency ranging pseudo-randomly 500–1200 ms); finally, another fixed, central target key must be depressed as fast as possible in response to this stimulus for 48 trials. To obtain an *initiation score*, we measured the latency for the subject to lift the finger off of the start key in response to the appearance of the stimulus on the screen.

Assessing the *planning* component requires a resource-demanding task where a choice action depends on the integration of several bits of information (Sorel and Pennequin, 2008; Toglia and Berg, 2013). In this task, the participant must correctly press one of two pseudo-randomly lateralized keys, contingent on the combination of two features of a central visual pattern stimulus: if the stimulus is blue or has horizontal stripes, the key on the left is correct; if the stimulus is orange or contains vertical stripes, the key on the right is correct. Each of the presented stimuli contained one of these features together with a second, non-contributing feature (e.g., vertical stripes that are green-colored). The influence of working memory confounds were minimized by making the choice patterns visually available for patients during performance. A *planning score* is generated by averaging the total latencies on correct trials.

To assess the *motivation* component, the participant performs the initiation task described above; here, participants are additionally given an amount of money in the form of monetary units at the beginning of the task, and money is taken away as a “penalty” if they do not press the target key more rapidly to a stimulus (triangle) relative to their previous performance. Participants receive both verbal and visual feedback (a bank of points appears on the screen) about their response speed after each trial on the computer screen compared to their reaction time during the initiation task, and participants are told that monetary units are converted to money at the end of the study. Participants also perform a “reward” condition where they receive points for responding more rapidly than during the initiation condition (reward and penalty conditions were administered in a randomly ordered manner across participants). In this study, we use the penalty condition to obtain a *motivation score* because previous work has shown that bvFTD patients are insensitive to negative feedback relative to positive feedback (Grossman et al., 2010). Unbeknownst to participants, all receive the same final amount for participation by adjusting the dollar value of a monetary unit.

### Neuroimaging data

Structural T1-weighted MRI data were available for all bvFTD participants with PACT scores (*n* = 18), and diffusion tensor imaging (DTI) data from the same scan session were available for a subset of participants (*n* = 15). High-resolution T1-weighted 3-dimensional spoiled gradient echo images were acquired on a Siemens 3.0T Trio scanner with an 8-channel coil (repetition time = 1620 ms, echo time = 3 ms, slice thickness = 1.0 mm, flip angle = 15°, matrix = 192 × 256, and in-plane resolution = 0.9 × 0.9 mm). Diffusion-weighted images (DWI) were acquired using a single-shot, spin-echo, diffusion-weighted echo planar imaging sequence (FOV = 245 mm; matrix size = 128 × 128; number of slices = 57; voxel size = 2.2 mm isotropic; TR = 6700 ms; TE = 85 ms; fat saturation). In total, 31 volumes were acquired per subject, one without diffusion weighting (*b* = 0 s/mm^2^) and 30 with diffusion weighting (*b* = 1000 s/mm^2^) along 30 non-collinear directions. We selected a sample of 24 demographically-matched imaging controls from our control panel with existing MRI and DTI data for neuroanatomical comparison as previously reported (Healey et al., 2015). Two sample *t*-tests confirmed that imaging controls [mean age = 60.71 years (SD = 6.9); mean education = 15.79 years (SD = 1.9)] are demographically comparable to patients (age, education, and gender, all *p* > 0.1).

#### Gray matter imaging

All images were preprocessed using PipeDream (https://sourceforge.net/projects/neuropipedream/) and Advanced Normalization Tools (ANTS, http://www.picsl.upenn.edu/ANTS/) to perform accurate, large-scale, multivariate normalization, as described (Avants et al., 2011a). Before normalization, each individual's structural image was segmented into tissue classes using *Atropos*, a voxel-based segmentation tool that segments the brain into GM, WM and cerebrospinal fluid (Avants et al., 2011b). A diffeomorphic deformation was used for registration that is symmetric so that it is not biased toward the reference space for computing the mappings (Avants et al., 2011a). Processing involved mapping T1 structural MRI to an unbiased average-shape and average-appearance template derived from a representative population consisting of 25 healthy seniors and 25 patients with FTD (Kim et al., 2008). This top-performing diffeomorphic method for registration and normalization avoids the need to use identical participants in the local template (Klein et al., 2009). GM probability images were calculated as a quantitative measure of GM density which allows us to generate a voxel-wise measurement and perform a voxel-based morphometric analysis of regional GM atrophy.

GM probability images were then transformed into Montreal Neurological Institute (MNI) space for statistical analysis and down-sampled to 2 mm^3^ resolution to attain a more anatomically relevant voxel size.

SPM8 (http://www.fil.ion.ucl.ac.uk/spm/software/spm8) was used to smooth GM images using a 5 mmFWHM Gaussian kernel. Smoothing was performed to minimize confounds associated with individual differences in structural anatomy. A whole brain analysis was conducted. First, GM density in bvFTD was compared with healthy seniors using a two-sample *t*-test with a voxel level threshold of *p* < 0.001 (false discovery rate [FDR]-corrected) and extent threshold of 50 voxels. In the next analysis, regressions were performed to relate GM density in bvFTD directly to the initiation, planning and motivation scores on the PACT. We also used regression to relate GM density in bvFTD to the NPI apathy FxS scores. Regression analyses were restricted to evaluating potential relationships between PACT and NPI performance within regions demonstrated to be atrophied in the bvFTD sample. This was done in an effort to constrain our interpretations of the regression analyses to those brain regions highly likely to have disease. For example, a significant correlation between a non-atrophied area and a PACT score could otherwise be attributed to factors that are independent of disease and instead related to non-specific factors such as age or other individual differences. The height threshold for the regression analyses was set at *p* < 0.005 (uncorrected). The threshold was set at *p* < 0.05 for the planning regression due to limited variance in planning scores. We accepted as significant a cluster with a volume of 30 adjacent voxels and a peak voxel Z-score > 3.09 (equivalent to *p* < 0.001).

#### White matter imaging

DWIs were preprocessed using ANTS software. Briefly, the unweighted (*b* = 0) images are first extracted and averaged. All DW images (including the individual *b* = 0 volumes) are then aligned to the average *b* = 0 using ANTs (Tustison et al., 2014). An affine transform is applied to capture eddy distortion in the DW images as well as motion. Diffusion tensors are computed using a weighted linear least squares algorithm in Camino (Cook et al., 2005). The corrected average *b* = 0 image is aligned to the subject's T1 image from the same scanning session, first rigidly to correct for motion, then using a deformable diffeomorphic transformation with mutual information to correct for inter-modality distortion. The diffusion to T1 warp is composed with the T1 to template warp (from the cortical thickness pipeline), producing a mapping from DWI space to the population T1 template in a single interpolation. Tensors are resampled into the template space using log-Euclidean interpolation (Arsigny et al., 2006) and reoriented to preserve the anatomical alignment of WM tracts (Alexander et al., 2001). We report fractional anisotropy (FA) that was computed on the tensor image in the group analysis template space. The resulting FA images were smoothed using a 4 mm FWHM isotropic Gaussian kernel.

DTI analyses of FA were performed in SPM8 using the two-samples *t*-test module. DTI volumes were analyzed using an explicit mask (FA > 0.25) in order to constrain comparisons to regions of WM. Comparisons of bvFTD participants to healthy seniors used a *p* < 0.005 (FDR-corrected) height threshold and a 200-voxel extent. Regression analyses were constrained to WM tracts with reduced FA using an explicit mask generated from the results of the direct comparison with healthy seniors. Our analyses were limited to WM tracts with significant disease, as above, in order to constrain our interpretation to disease-specific neuroanatomical regions. For these regression analyses we accepted as significant a cluster with a volume of 150 adjacent voxels and a peak voxel Z-score > 3.3 (equivalent to *p* < 0.0005).

## Results

### Behavioral data results

Table 2 summarizes the performance on the PACT measures. Between-group comparisons found that apathetic bvFTD patients have slower latencies than normal controls on all three measures of GDB: Initiation [*t*_(33)_ = 2.26, *p* = 0.03]; Planning [*t*_(33)_ = 4.79, *p* < 0.001]; Motivation [*t*_(33)_ = 2.17, *p* = 0.03]. We found a significant correlation between initiation and motivation performance (*r* = 0.78; *p* < 0.001). However, correlations are not significant between other PACT measures (all *p* > 0.05, Bonferroni corrected)

**Table 2:** **Mean (S.D.) reaction time scores for PACT performance**.

**PACT score**	**Control (*n* = 17)**	**bvFTD (*n* = 18)**	***p*-value**
Initiation	364.2 ms ± 54.0	587.50 ms ± 404.3	0.03
Planning	1023.76 ms ± 139.9	1754 ms ± 612.5	< 0.001
Motivation	522.31 ms ± 113.6	916 ms ± 715.5	0.03

Mean apathy FxS score on the NPI for the bvFTD group was 5.27 ± 3.3. Mean caregiver distress associated with apathy was 2.77 ± 1.4. Caregiver distress scores and FxS scores were moderately correlated (*r* = 0.53; *p* = 0.03).

### Imaging results

#### Gray matter imaging

**Figure 1:**
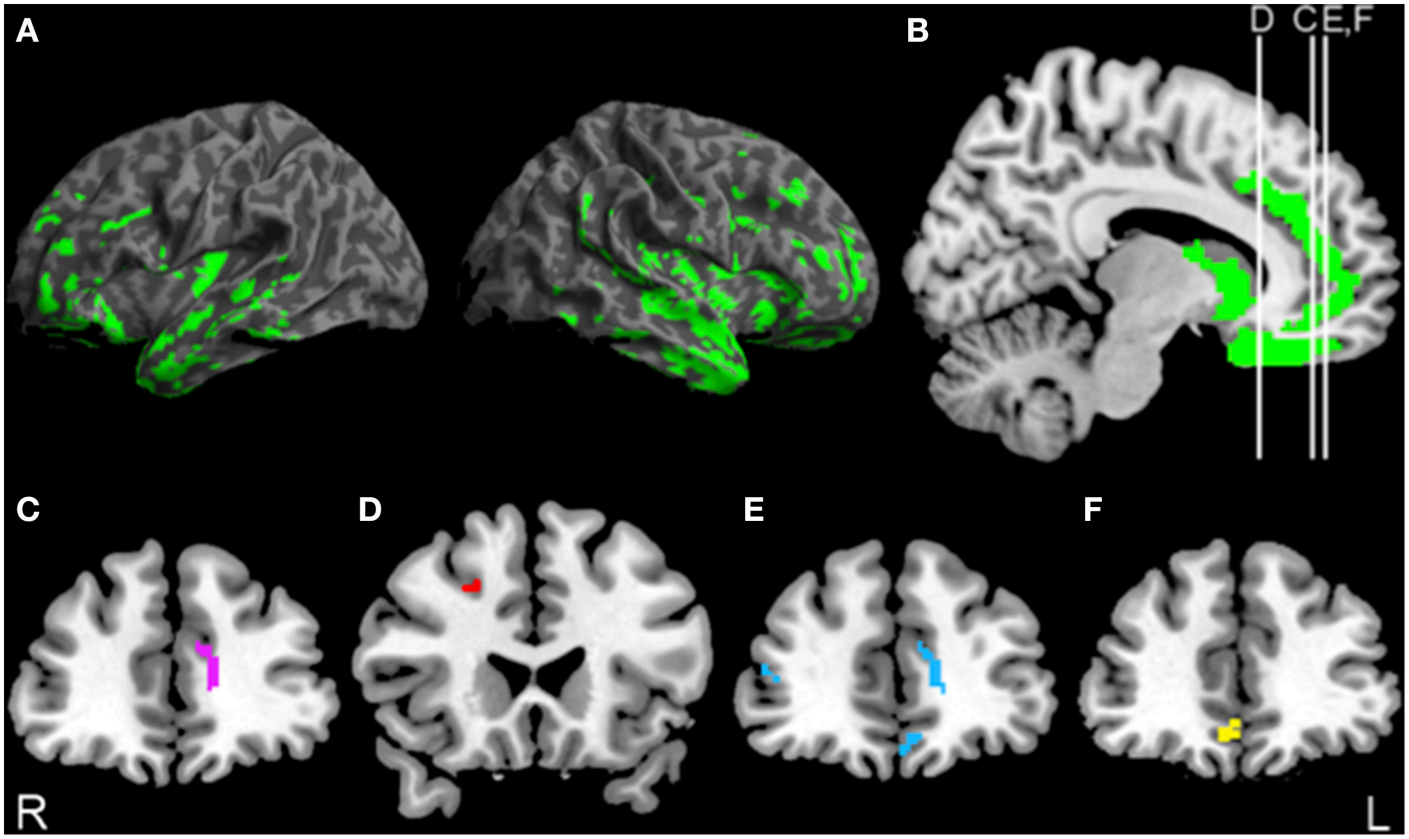
**Significant atrophy in bvFTD, and regressions relating PACT performance to gray matter density. (A,B)** Anatomic distribution of significant gray matter atrophy in patients with behavioral variant frontotemporal degeneration (green). **(C)** Significant regression relating initiation performance to cortical atrophy in anterior cingulate (purple) illustrated at *y* = 40. **(D)** Significant regression relating planning performance to cortical atrophy in dorsolateral prefrontal cortex (red) illustrated at *y* = 22. **(E)** Significant regression relating motivation performance to cortical atrophy in orbitofrontal cortex (blue) illustrated at *y* = 42. **(F)** Significant regression relating NPI apathy FxS scores to cortical atrophy in orbitofrontal cortex (yellow) illustrated at *y* = 40. See text and Supplemental Table S1 for details.

Figure 1 illustrates reduced GM density (green) in lateral (Figure 1A) and medial (Figure 1B) frontal and temporal regions in bvFTD compared to controls. The Supplemental Table S1 summarizes the coordinates of peak voxels in significantly atrophic clusters.

The results of the regression analysis relating PACT performance to reduced GM density are illustrated in Figure 1 as well. Initiation performance was related to anterior cingulate (ACC) (Figure 1C, purple). Planning performance was related to dorsolateral prefrontal cortex (dlPFC) (Figure 1D, red). Motivation performance was related to orbitofrontal cortex (OFC) and Anterior Cingulate Cortex (ACC) (Figure 1E, blue). Voxel coordinates for these regressions are summarized in the Supplemental Table S1. NPI apathy scores were related only to OFC (Figure 1F, yellow).

#### White matter imaging

**Figure 2:**
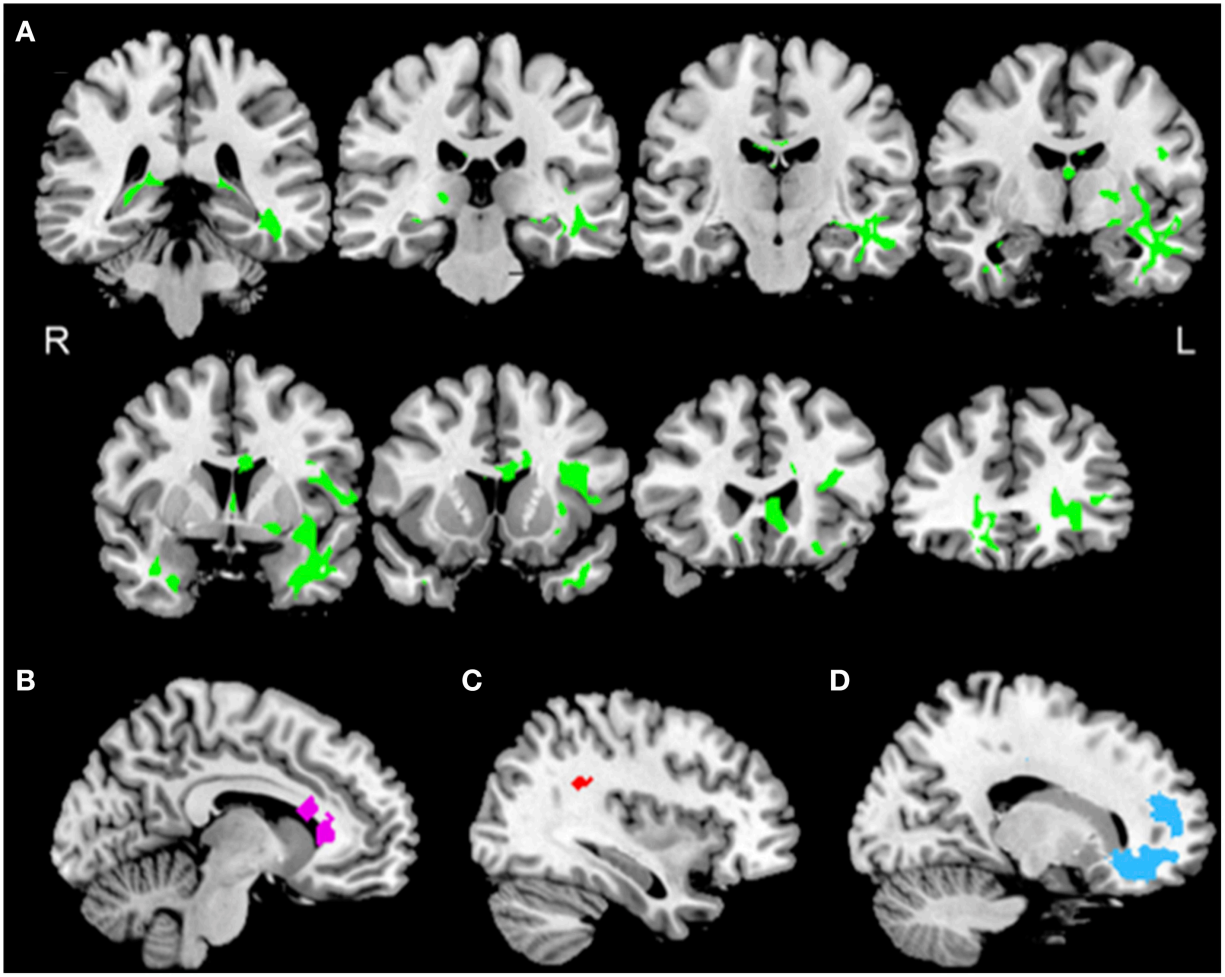
**Reduced white matter integrity in bvFTD, and regressions relating PACT performance to reduced FA. (A)** Anatomic distribution of reduced fractional anisotropy in patients with behavioral variant frontotemporal degeneration (green). **(B)** Significant regression relating initiation performance to reduced FA including cingulum (purple) illustrated at *x* = −9. **(C)** Significant regressions relating planning performance to reduced FA including right superior longitudinal fasciculus (red) illustrated at *x* = 35. **(D)** Significant regressions relating motivation performance to reduced FA in uncinate fasciculus and prefrontal corona radiata (blue) illustrated at *x* = 16. See text and Supplemental Table S1 for details.

bvFTD showed reductions in FA in bilateral frontal and temporal WM relative to controls (Figure 2A, green). Coordinates of peak voxels in clusters of significantly reduced FA, and regressions of FA with PACT scores are summarized in the Supplemental Table S1. Initiation performance was related to FA in cingulum, uncinate fasciculus (UNC), inferior longitudinal fasciculus (ILF), and corpus callosum (CC) (Figure 2B, purple). Planning performance was related to FA in superior longitudinal fasciculus (SLF), inferior frontal-occipital fasciculus (IFO), and rostral frontal corona radiata (CR) and CC, as well as posterior thalamic radiations (Figure 2C, red). Finally, motivation performance was related to FA in UNC as well as CC, CR, and ILF (Figure 2D, blue).

## Discussion

This study investigated the behavioral and neural basis of GDB by examining patients with bvFTD. These patients display prominent apathy and therefore bvFTD is a valuable group for investigating apathy. We found that apathetic bvFTD patients are impaired on each of the three processes thought to contribute to the impairments in GDB that underlie apathy, including initiation, planning and motivation. These three GDB processes were associated with disease in three distinct frontal GM regions and in WM projections between these regions and other brain areas (Massimo et al., 2009).

To our knowledge, this is the first study to use an objective behavioral assessment of apathy. In previous studies, apathy was ascertained by querying patient caregivers using questionnaires. Unfortunately, this may be confounded in part by the impact of caregiver stress on judgments of apathy (Boyer et al., 2004). Other studies use patient reports of apathy (Cacciari et al., 2010). Due to limited self-appraisal in bvFTD (Massimo et al., 2013), it is not reliable to ask bvFTD patients directly about their perception of their own apathy. Further, beyond confirming the presence of apathy, current instruments such as the NPI are ineffective in identifying different subtypes of apathy (Chow et al., 2009). We did not find correlations between PACT scores with either NPI caregiver-distress scores or NPI FxS apathy scores. This lack of finding provides support for the notion that the NPI is not sensitive to the full spectrum of behaviors associated with apathy.

Therefore, in the present study, we developed a novel reaction time measure that directly ascertains each of the three components thought to play a role in GDB in bvFTD. We discuss below behavioral aspects of apathy and the neuroanatomic associates of these impairments.

### Behavioral deficits contributing to apathy

The PACT identified an impairment in each of the three components of GDB that we ascertained. GDB is a complex process that includes many components, but we focused on three components thought to play a central role in the emergence of apathy, including initiation, planning, and motivation. From the perspective of our GDB model, a deficit in any one of these components can result in apathy in bvFTD.

Consider first a deficit in initiation. Initiation depends in part on a signal that is sufficiently strong to begin an action. We found a deficit in initiation in bvFTD. We are not aware of previous assessments of initiation in these patients. In our assessment, we did not use a simple reaction time test to measure initiation because of the two components involved in this kind of task. Thus, simple reaction time involves both starting an action in response to a stimulus and stopping the timing clock by completing an action. We were specifically interested in the first of these components. We do not think that performance was confounded by perceptual difficulty such as noticing the initiating signal because there were no other competing signals, and patients were not otherwise distracted during task performance. While bvFTD can be associated with motor weakness as in amyotrophic lateral sclerosis (Burrell et al., 2011), the patients participating in this study had no motor deficits that could have confounded performance. We also do not believe that performance was confounded by some other cognitive component because the signal was maximally simplified to involve only the appearance of the stimulus. There were no associated choices or decisions that were required—merely lifting a finger to start an action in response to the appearance of a single stimulus. Using this simple task, we demonstrated that initiation is significantly compromised in bvFTD.

We also found that patients with bvFTD have limitations in a planning component of GDB that can contribute to apathy. In this component of the PACT, patients had to selectively detect the presence of one of two features of a stimulus, and then associate this with a left- or right-lateralized response key. bvFTD patients were significantly slowed in their performance on this task. In a *post-hoc* analysis, we observed an inverse correlation between our measure of planning and number of responses on letter guide-fluency (*r* = −0.46, *p* = 0.03), a task that requires selection and planning of phonological category information (Birn et al., 2010). It was challenging to develop a measure of planning that is more clearly representative of organization that is also compatible with a discrete reaction time that can be associated with GDB. For example, patients with bvFTD are impaired on measures like Tower of London (Franceschi et al., 2011), but it is difficult to assess discrete reaction times when using an obviously multi-component strategic task like this. Additional work will be needed to develop other tasks that may better capture the planning component of GDB. Nevertheless, patient studies using caregiver questionnaires have found a relationship between apathy and poor executive function in bvFTD (Zamboni et al., 2008; Eslinger et al., 2012). Eslinger and colleagues found that caregiver judgments of bvFTD patients' apathy was significantly correlated with executive function measures, suggesting that apathy emanates in part from difficulty manipulating and integrating elements of a plan to perform an action (Eslinger et al., 2012). Our findings are compatible with this observation.

We observed a deficit in the motivation component of GDB as well. Patients' response latencies did not improve even though we “punished” them by taking money away from a pot of funding that they were given at the beginning of the task. Their performance in response to a reward improved in a manner that did not differ from controls, emphasizing the patients' ability to perform various aspects of the task such as appreciating the numerical component and increments in this as a function of performance. Similarly, it is unlikely that reaction time performance was at ceiling because they were able to respond more rapidly under the reward condition. Deficits in processing value associated with an action have been examined previously in patients with bvFTD because they appear to have early degeneration of a frontal reward circuit in comparison to other neurodegenerative conditions (Rabinovici et al., 2007). Poor motivation can occur in these patients because they have decreased reactivity to positive “reward” and negative “punishment” signals, thereby making goal-selection difficult (Levy and Dubois, 2006). Experimental evidence, however, has emphasized that patients with bvFTD and other diseases affecting OFC have the greatest difficulty interpreting “punishment” signals (Grossman et al., 2010). Impaired processing of negatively valenced emotional stimuli, such as failure to recognize anger or sadness in others, can contribute to poor empathy (Kipps et al., 2009) which has also been associated with disease in the OFC in FTD (Kamminga et al., 2015). In another study of empathy in bvFTD (Eslinger et al., 2011), the perspective-taking component of empathy was related to lateral bifrontal disease while the emotional component of empathy was related to a medial frontal region. Clinically, patients with bvFTD do not appear to be responsive to negative feedback when caregivers try to modify their inappropriate behaviors. Even though money appeared to be an effective reward, it is possible that withdrawal of money may not have been the optimal form of punishment, and future work can examine whether withdrawal of other valuable stimuli such as food or emotion may prove more effective at motivating performance.

### Anatomic basis for apathy in bvFTD

We found that difficulty with initiation is associated with atrophy of ACC. Considerable work has suggested that the ACC is important for initiating behavior (Tekin and Cummings, 2002). The ACC has previously been implicated in processes that influence action initiation in healthy adult studies (Mulert et al., 2003). The ACC is also implicated in initiation difficulty in those with frontal lobe injury. The *akinetic mute state* describes patients who tend to sit quietly in the same position all day without speaking or talking, and this has been specifically related to ACC damage (Mega and Cohenour, 1997). The ACC has been well studied in dementia, and neuroimaging evaluations have linked the ACC region to apathy in various groups. Reduced GM density in the cingulate gyrus has been associated with apathy in patients with bvFTD (Zamboni et al., 2008; Massimo et al., 2009) and PD (Reijnders et al., 2010).

We also found that the cingulum, a WM pathway associated with ACC, is also related to impaired initiation. This projection relates projections from the cingulate region with other areas implicated in GDB. Previous DTI studies investigating WM disease and apathy also have shown an association with the cingulum, which has reciprocal connections between ACC and the medial orbitofrontal region that is important for motivation (Hahn et al., 2013). In healthy adults, ACC and dlPFC structures work in concert during complex tasks that require attentional control, and this is likely to be mediated through the cingulum (Silton et al., 2010). Supplemental motor areas important for the execution of action also exchange projections with the cingulate gyrus via the cingulum. Interruption of projections within the large-scale neural network subserving GDB, such as projections between ACC and other structures important for GDB, thus may be contributing to apathetic behavior. Fibers in UNC may help integrate ACC and the amygdala in the anterior temporal lobe that is an important structure for GDB and CC is likely to help integrate initiation processes supported by ACC across the two hemispheres.

Deficits in the planning component of GDB were associated with atrophy in the dlPFC and reduced FA in related WM tracts, including SLF and frontal CR. fMRI studies of healthy adults suggest that dlPFC contributes to planning (Di et al., 2013). Imaging studies of patients with FTD and Alzheimer's disease (AD) have linked apathetic behavior on the caregiver-rated questionnaires to atrophy in dlPFC as well (Zamboni et al., 2008; Massimo et al., 2009).

The SLF is a prominent WM tract interconnecting the frontal, temporal, and parietal lobes, and this tract has been implicated in the integration of these diverse regions involved in planning (Genova et al., 2013). A previous study of patients with amnestic mild cognitive impairment (aMCI) revealed a relationship between reduced FA in the SLF and apathy on patient-based ratings of apathy (Cacciari et al., 2010). Our findings suggest that planning necessary for GDB may be compromised following disease in dlPFC and associated WM tracts linking this region with other brain areas. Fibers in CR are likely to play a role in integrating function across OFC and dlPFC, and CC fibers may mediate planning processes across the two hemispheres.

We found that difficulty with the motivation component of GDB is associated with atrophy in OFC and related WM tracts, including UNC. Evidence from healthy subject fMRI studies suggests that OFC plays a role in interpreting value and reward-related information (Hare et al., 2010). Imaging evidence from patients with bvFTD has emphasized the link between OFC and apathetic behavior (Massimo et al., 2009). FDG-glucose PET brain activity is decreased in OFC in bvFTD patients with apathy compared to non-apathetic patients (Peters et al., 2006). OFC, has also been previously associated with disinhibited behaviors in FTD and this may be related in part to this region's role in generating adequate responses to environmental changes (i.e., patients with OFC damage fail to adapt behavior flexibly; Hornberger et al., 2011). It is important to point out that the apathy scale of the NPI is correlated only with OFC atrophy. This suggests that the NPI, while measuring a component of apathy, is not adequately sensitive to the full spectrum of behaviors associated with apathy. Inspection of the questions probing apathy on the NPI in fact are highly oriented toward probes of motivation (e.g., “Has you family member lost interest in doing things or lacks the motivation to start new activities?”), without adequate sensitivity to initiation or planning.

UNC is a major tract connecting the anterior temporal lobe with the medial and lateral ventral prefrontal cortex areas known to be important for GDB (Kable and Glimcher, 2007). The amygdala in the anterior temporal lobe is an important structure that may contribute to motivation (Jiang et al., 2014). DTI studies performed in Alzheimer's disease (AD) and Progressive Supranuclear Palsy (PSP) implicated UNC in apathy (Hahn et al., 2013). Thus, our findings also underline that disease in OFC and associated WM tracts can interfere with the motivational component of apathy. Projections in the CR are likely to link OFC with dlPFC, and fibers in CC are likely to integrate motivational mechanisms in the two hemispheres.

While we suggest specific contributions of neural mechanisms to distinct components of GDB, we do observe some overlap across measures. For example, our GM observations suggest that the cingulate may contribute to both initiation and motivation. This is not a surprising finding given the role of the ACC in generating response to punishment cues (Shinagawa et al., 2015). Additionally, we observed a significant correlation between initiation and motivation performance (*r* = 0.78; *p* < 0.001). Future research that further differentiates these two components of GDB may help elucidate the specific role of the ACC in apathy. Our failure to find significant correlations between other PACT measures is otherwise consistent with our observation of distinct neuroanatomical regions contributing to components of GDB.

One goal of the present study was to demonstrate an empirically-based approach to elucidating mechanisms contributing to apathy. This work is the first step in the development of an instrument that would be based on objective, empirical measurements of impairments of each of the components of GDB that contribute to apathy. Such an instrument would improve on the current instruments because of its objective basis, its sensitivity to distinct components of apathy, and its ability to increase the likelihood of detecting and targeting treatment of specific subtypes of apathy. Apathy constitutes one of the six diagnostic criteria for bvFTD (Rascovsky et al., 2011) and the PACT could potentially provide an objective assessment of this clinical feature. Our findings also have potentially important implications for its treatment. Unfortunately, prior interventions to manage apathy have not been effective (Mizrahi and Starkstein, 2007). One reason for this failure may be the way in which apathy is conceptualized. That is, apathy is largely viewed homogeneously, as if derived from a single source, such as poor motivation. For example, the most commonly used instrument to measure apathy, the NPI, primarily ascertains diminished motivation. Interventions targeting only one component of apathy may not be addressing the specific component of GDB that is compromised in a particular patient. One goal of the present study was to quantify comprehensively multiple components of GDB in apathetic participants in an objective manner. This would allow investigators to obtain a more appropriate perspective on apathy since our findings suggest that each of three components of GDB contribute to apathetic behavior. Moreover, these components appear to be relatively independent since each of these components is associated with a relatively distinct GM and WM regions, and interventions may need to address all components of apathy.

Some limitations should be kept in mind when considering our findings. We used the NPI to determine the presence of apathy. Future studies should include other scales such as Frontal Systems Behavior Scale (Malloy et al., 2007), which may be more sensitive for assessing apathy. Although our sample was larger than in prior investigations of apathy, we nevertheless studied a relatively small number of patients and power in the imaging studies may not have been sufficient to detect every anatomic region associated with apathy. Because floor effects in performing the planning measure limited variance, we were forced to use a more liberal threshold for our GM analyses. Lastly, we do not have neuropathological confirmation of the diagnoses of these patients.

With these caveats in mind, we conclude that apathetic behavior in bvFTD can be characterized as a multi-component impairment in GDB that may compromise processes including initiation, planning and motivation. These three processes are supported by a large-scale neural network constituting the neuroanatomic basis for GDB, including distinct GM regions within the frontal lobe and related WM projections.

## Author contributions

LM, the corresponding author, is responsible for drafting the manuscript, study concept and design, analysis and interpretation of data, acquisition of data, statistical analysis and obtaining funding. LM reports no disclosures. JP is responsible for interpretation of data and acquisition of data. JP reports no disclosures. LE is responsible for revising the manuscript, study design, interpretation of data and study supervision. LE reports no disclosures. CM is responsible for revising the manuscript and interpretation of data. CM reports no disclosures. KR is responsible for study design and interpretation of data. KR reports no disclosures. PE is responsible for interpretation of data. PE reports no disclosures. ME is responsible for revising the manuscript and interpretation of data. ME reports no disclosures. DI is responsible for interpretation of data. DI reports no disclosures. MG is responsible for revising the manuscript, study design, interpretation of data, study supervision and obtaining funding. MG reports no disclosures.

### Conflict of interest statement

The authors declare that the research was conducted in the absence of any commercial or financial relationships that could be construed as a potential conflict of interest.
