# Supplementary material for: Apathy in Frontotemporal Degeneration: Neuroanatomical Evidence of Impaired Goal-directed Behavior
Source: Front Hum Neurosci. 2015 Nov 10;9:611. doi: 10.3389/fnhum.2015.00611 (PMC4639601; doi:10.3389/fnhum.2015.00611)
Supplement: Supplementary file 1 [file Table1.DOCX]

Supplemental Table S-1: Anatomic Locus of Peak Voxels in Clusters Relating PACT Scores to Grey Matter Atrophy (n=18) and White Matter Integrity in bvFTD (n=15)

| Anatomic Locus (BrodmannArea)^1^ | | MNI Coordinates^2^ | | | | Z-Score of Peak Voxel | | Cluster Size (voxels) | | |
| --- | --- | --- | --- | --- | --- | --- | --- | --- | --- | --- |
|  |  | **X** | | **Y** | **Z** |  |  |  |  |  |
| **bvFTD<Eld (GM atrophy)** | | | | | | | | | | |
| L superior frontal gyrus (10) | | -22 | | 46 | 26 | 4.65 | | 96 | | |
| R rostral prefrontal (11) | | 22 | | 52 | 4 | 5.17 | | 362 | | |
| R middle frontal gyrus (9) | | 20 | | 26 | 38 | 5.06 | | 106 | | |
| R inferior frontal gyrus (44) | | 40 | | 8 | 28 | 5.84 | | 401 | | |
| L insula | | -22 | | 20 | -6 | 4.69 | | 194 | | |
| R subcallosal gyrus (25) | | 16 | | 18 | -8 | 5.24 | | 524 | | |
| R parahippocampal gyrus (27) | | 18 | | -34 | -2 | 6.25 | | 14067 | | |
| R fusiform gyrus (20) | | 40 | | -34 | -20 | 4.51 | | 99 | | |
| R middle temporal gyrus (20) | | 58 | | -32 | -18 | 4.70 | | 94 | | |
| R inferior temporal gyrus (37) | | 54 | | -52 | -10 | 4.90 | | 149 | | |
| R inferior parietal lobule (40) | | 34 | | -34 | 38 | 5.10 | | 64 | | |
| **bvFTD Initiation Regression (GM)** | | | | | | | | | | |
| R dorsal anterior cingulate gyrus (32) | | 22 | | 16 | 42 | 4.87 | | 52 | | |
| L dorsal anterior cingulate gyrus (32) | | -14 | | 42 | 14 | 4.30 | | 74 | | |
| **bvFTD Planning Regression (GM)** | | | | | | | | | | |
| R middle frontal gyrus (9) | | 22 | | 14 | 44 | 3.28 | | 56 | | |
| L middle frontal gyrus (11) | | -20 | | 40 | -22 | 3.10 | | 104 | | |
| **bvFTD Motivation Regression (GM)** | | | | | | | | | | |
| L medial orbital frontal gyrus (11) | | -4 | | 44 | -16 | 4.61 | | 42 | | |
| R inferior frontal gyrus (46) | | 40 | | 38 | 10 | 3.90 | | 42 | | |
| R inferior frontal gyrus (47) | | 34 | | 34 | 2 | 3.17 | | 78 | | |
| L inferior frontal gyrus (47) | | -48 | | 24 | -6 | 3.52 | | 34 | | |
| R cingulate gyrus (32) | | 22 | | 18 | 40 | 5.41 | | 63 | | |
| L cingulate gyrus (32) | | -14 | | 42 | 14 | 4.16 | | 77 | | |
| **bvFTD NPI Apathy FxS regression (GM)** | | | | | | | | | | |
| R. medial orbital frontal gyrus (11) | 4 | | 38 | | -12 | | 3.99 | | | 56 |
| R. middle frontal gyrus (11) | 26 | | 30 | | -18 | 3.25 | | | 68 | |
| **bvFTD<Eld (reduced FA)** | | | | | | | | | | |
| L uncinate fasciculus | | -33 | | 2 | -9 | 6.30 | | 13222 | | |
| R uncinate fasciculus | | 38 | | 7 | -28 | 5.15 | | 1388 | | |
| L inferior frontal gyrus white matter | | -34 | | 14 | 22 | 5.87 | | 7623 | | |
| R cingulum | | 3 | | -21 | 30 | 4.60 | | 355 | | |
| R anterior corona radiata | | 11 | | 34 | -13 | 4.54 | | 1244 | | |
| Body of corpus callosum | | -7 | | 6 | 25 | 4.44 | | 1546 | | |
| R column and body of fornix | | 3 | | -8 | 16 | 6.42 | | 541 | | |
| L posterior limb of internal capsule | | -18 | | -8 | 5 | 4.17 | | 347 | | |
| L crus of fornix or striaterminalis | | -14 | | -30 | 13 | 4.76 | | 261 | | |
| Splenium of corpus callosum | | 12 | | -33 | 11 | 5.15 | | 1220 | | |
| R inferior temporal gyrus white matter | | 47 | | -48 | -13 | 5.10 | | 335 | | |
| **bvFTD Initiation Regression (FA)** | | | | | | | | | | |
| L cingulum | | -7 | | 30 | 15 | 3.43 | | 1693 | | |
| Body of corpus callosum | | 2 | | 8 | 25 | 3.52 | | Same cluster as cingulum | | |
| Genu of corpus callosum | | 13 | | 52 | 14 | 4.03 | | 1056 | | |
| Genu of corpus callosum | | -12 | | 53 | 23 | 3.39 | | 382 | | |
| R uncinate fasciculus | | 16 | | 39 | -16 | 4.84 | | 2587 | | |
| L medial orbital gyrus white matter | | -14 | | 32 | -16 | 4.29 | | 4609 | | |
| **bvFTD Planning Regression (FA)** | | | | | | | | | | |
| R superior longitudinal fasciculus | | 25 | | -40 | 34 | 3.47 | | | 191 | |
| L inferior frontal gyrus white matter | | -49 | | 31 | 5 | 3.54 | | | 217 | |
| R inferior frontal occipital fasciculus | | 18 | | 25 | -3 | 5.10 | | | 1092 | |
| Genu of corpus callosum | | 12 | | 47 | 26 | 4.23 | | | 650 | |
| Body of corpus callosum | | 15 | | 15 | 42 | 3.82 | | | 533 | |
| Body of corpus callosum | | 10 | | 6 | 58 | 3.74 | | | 162 | |
| R posterior corona radiata | | 20 | | -26 | 37 | 3.93 | | | 258 | |
| L superior corona radiata | | -20 | | -14 | 38 | 3.54 | | | 269 | |
| L. cingulum | | -5 | | -8 | 37 | 3.45 | | | 239 | |
| **bvFTD Motivation Regression (FA)** | | | | | | | | | | |
| R uncinate fasciculus | | 16 | | 39 | -16 | 4.67 | | | 2537 | |
| L medial orbital gyrus white matter | | -13 | | 33 | -16 | 3.98 | | | 1678 | |
| Genu of corpus callosum | | 16 | | 51 | 15 | 3.92 | | | 1091 | |
| Genu of corpus callosum | | 2 | | 25 | 7 | 3.77 | | | 2593 | |

NOTES:

1. The corresponding Brodmann area is indicated by the figure in parentheses. L=left; R=right. 2. Peak locus of these clusters are derived from MNI space converted to Talairach space using MNI.
